# Length matters: the disordered N-terminus of Pal coordinates Lpp exclusion for outer membrane constriction in *E. coli*

**DOI:** 10.1128/jb.00408-25

**Published:** 2025-12-23

**Authors:** Zhuo-Wei Chen, Ting-Ting Chen, Hong-Su Zhang, Si-Yu Chen, Yu-Qing Zhang, Xiu-Lan Chen, Yu-Zhong Zhang, Hai-Nan Su

**Affiliations:** 1 Marine Biotechnology Research Center, State Key Laboratory of Microbial Technology, Shandong University 520252, Qingdao, China; 2 MOE Key Laboratory of Evolution and Marine Biodiversity, Frontiers Science Center for Deep Ocean Multispheres and Earth System and College of Marine Life Sciences, Ocean University of China 670391 , Qingdao, China; 3 Laboratory for Marine Biology and Biotechnology, Qingdao Marine Science and Technology Center and Laoshan Laboratory 474988, Qingdao, China; University of Southern California, Los Angeles, California, USA

**Keywords:** outer membrane, lipoproteins, disordered region, cell division, Pal, Lpp

## Abstract

The peptidoglycan-associated lipoprotein (Pal) is essential for outer membrane integrity in gram-negative bacteria, but how its N-terminal disordered region contributes to cell division remains unclear. Here, we truncated this region in *Escherichia coli* (*pal*^−8^: 8-aa deletion, *pal*^−24^: 24-aa deletion) and found that while neither truncation impaired Pal localization or growth, both mutants exhibited graded sensitivity to membrane stressors (1 mM EDTA/1% SDS), with *pal*^−24^ showing intermediate susceptibility between *pal*^−8^ and Δ*pal*. Atomic force microscopy revealed that the disordered region length fine-tunes Pal’s ability to exclude Braun’s lipoprotein (Lpp) from division sites: *pal*^−8^ maintained wild-type Lpp-exclusion zones, whereas *pal*^−24^ exhibited either absent or narrowed grooves. These results suggest that the disordered region acts as a length-dependent molecular spacer, ensuring efficient Pal-Lpp spatial exclusion during cell division. Our work establishes a mechanistic paradigm by which Pal’s flexible N-terminus coordinates outer membrane constriction through dynamic competition with Lpp, offering new insights into the functional plasticity of disordered domains in bacterial morphogenesis.

**IMPORTANCE:**

The outer membrane of bacteria like *E. coli* must constrict during division, but the full mechanism is unclear. We studied peptidoglycan-associated lipoprotein (Pal), a protein that helps coordinate this process. Pal has a disordered linker region, and we found that the length of this linker acts as a molecular ruler. Shortening the linker disrupts Pal’s ability to properly organize the division site and create a zone required for outer membrane remodeling. This reveals how a seemingly unstructured protein region can perform a precise, measurement-dependent function. Since disordered linkers are common in bacterial outer membrane lipoproteins, our work suggests a general design principle for fine-tuning cell envelope dynamics during growth and division.

## INTRODUCTION

The envelope of gram-negative bacteria is a sophisticated tripartite structure comprising the outer membrane, peptidoglycan layer, and inner membrane (1). This architecture not only confers essential physiological functions but also demands precise spatial and temporal regulation during bacterial growth and division (2). While the constriction of the inner membrane and peptidoglycan layer is mediated by well-characterized systems like the FtsZ-driven divisome (3, 4), the mechanistic details of outer membrane invagination remain less understood. Current evidence points to a pivotal role of the peptidoglycan-associated lipoprotein (Pal) in this process (5–8).

Pal orchestrates outer membrane constriction through a dynamic interplay with Braun’s lipoprotein (Lpp) (5, 9). Both proteins interact with the peptidoglycan layer via meso-diaminopimelic acid, yet through fundamentally distinct mechanisms: Pal forms reversible non-covalent bonds (10), whereas Lpp establishes irreversible covalent cross-links (11). High-resolution atomic force microscopy (AFM) studies have recently revealed that during division, Pal recruitment to mid-cell creates an Lpp-exclusion zone at the division site, a spatial competition critical for proper outer membrane remodeling (9). Intriguingly, Δ*pal* mutants maintain division capability, with Lpp compensating by occupying the vacated peptidoglycan-binding sites (9), albeit at the cost of increased outer membrane vesiculation (12).

Structurally, Pal consists of an N-terminal lipid anchor embedded in the outer membrane, a disordered linker region (residues 25–68 of the precursor protein), and a C-terminal peptidoglycan-binding domain (13). This architecture reflects a conserved feature in *Escherichia coli*, where bioinformatic analyses reveal that half of outer membrane lipoproteins possess disordered linkers (14). While these regions have recently been shown to govern lipoprotein trafficking through the Lol system, Pal is still largely targeted to the outer membrane even with substantial linker truncation (Δ26–56, ~85% outer membrane localization), distinguishing it from other lipoproteins like RcsF and NlpD (14). While El Rayes et al. demonstrated that deletion of residues 26–56 caused only minor impairment in Pal’s outer membrane localization compared to other lipoproteins (14), its role in mediating interactions with other proteins, particularly Lpp, has not been investigated. Understanding the function of this disordered region could provide insights into how Pal coordinates with Lpp to maintain outer membrane integrity during cell division.

In this study, we aimed to elucidate the role of the N-terminal disordered region of Pal in *E. coli* cell division and its spatial competition with Lpp. Through targeted truncations (*pal*^−8^: ΔK26-S33, *pal*^−24^: ΔK26-N49), we dissected how progressive shortening affects Pal-Lpp spatial competition during division. Our findings reveal that while dispensable for basal localization, the disordered linker length fine-tunes Pal’s ability to exclude Lpp from division sites, providing direct evidence that these regions serve as molecular spacers in envelope morphogenesis.

## RESULTS

### Construction of Pal truncation mutants

**Fig 1:**
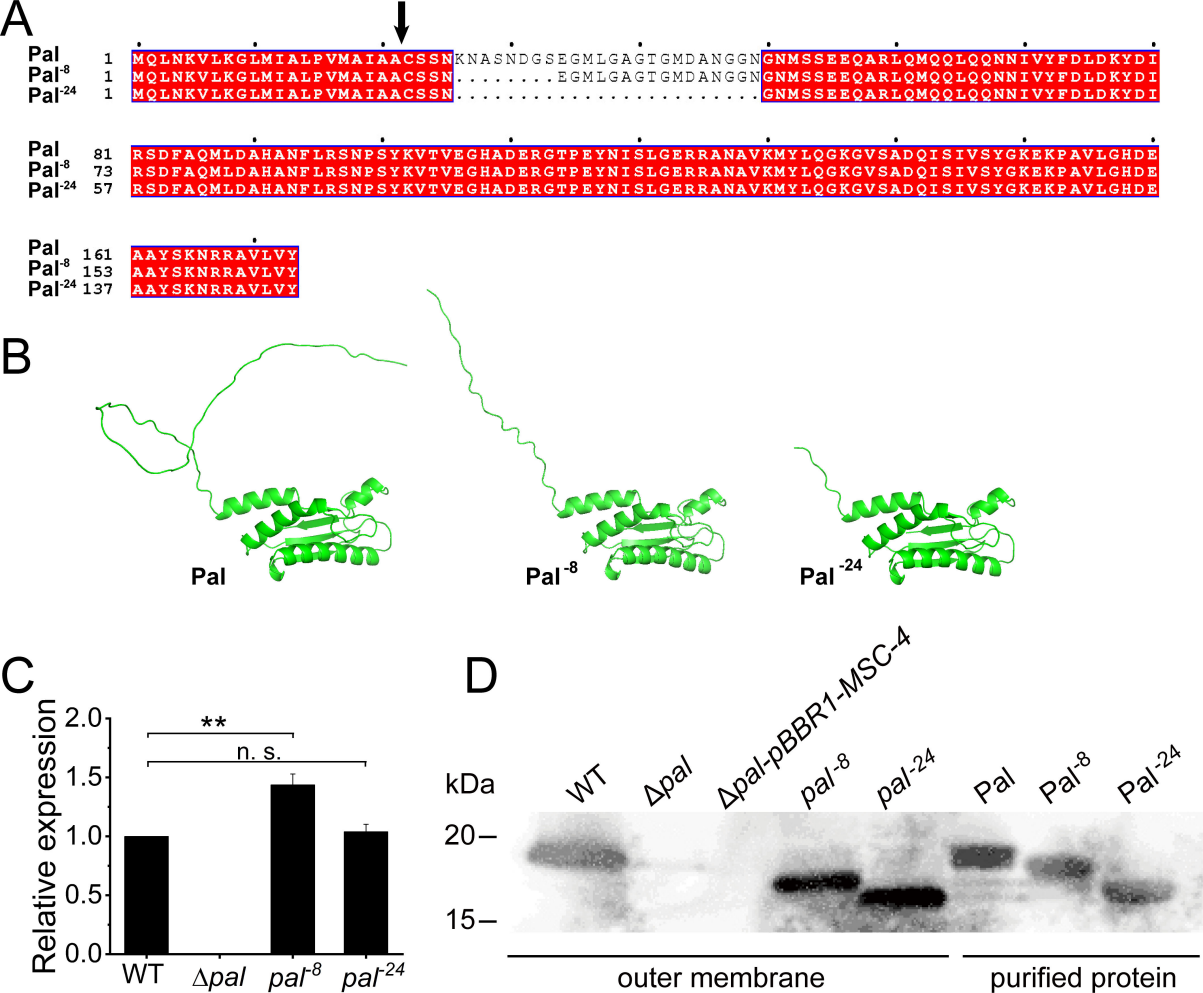
Construction and validation of Pal truncation mutants. (**A**) Amino acid sequence alignment of the precursor forms of wild-type (WT) Pal and the truncation mutants Pal^−8^ (ΔK26–S33) and Pal^−24^ (ΔK26–N49). The black arrowhead indicates the signal peptide cleavage site. (**B**) Predicted protein structures of WT Pal, Pal^−8^, and Pal^−24^, generated using AlphaFold3 (15). The overall fold of the C-terminal domain is preserved in the truncation mutants. (**C**) RT-qPCR analysis of *pal* gene expression in the indicated strains after 12-h cultivation. Data are presented as mean ± SD from three independent replicates. Statistical significance was determined by Student’s *t*-test. **P < 0.01. n.s., not significant. (**D**) Immunoblot analysis of Pal proteins in outer membrane fractions isolated by sucrose density gradient centrifugation. Purified recombinant proteins serve as migration controls and confirm antibody specificity.

To investigate the functional role of the N-terminal disordered region in the Pal protein, we constructed two truncation mutants: Pal^−8^ (deletion of amino acids between K26 and S33) and Pal^−24^ (deletion of 24 amino acids between K26 and N49) (Fig. 1A). Structural predictions using AlphaFold3 revealed that both Pal^−8^ and Pal^−24^ retained a folding pattern highly similar to that of the wild-type (WT) Pal protein (Fig. 1B). This suggested that the truncation of the disordered region did not disrupt the overall structure of Pal.

### Expression and localization of truncated Pal variants

To determine whether the truncation of the disordered region affected the expression of the *pal* gene, we performed RT-qPCR analysis. The results revealed that both *pal*^−8^ and *pal*^−24^ mutants maintained wild-type levels of *pal* gene transcription (Fig. 1C), indicating that the deletions did not interfere with gene expression regulation, while no *pal* gene transcription was detected in the Δ*pal* mutant (Fig. 1C).

To assess whether the truncation of the disordered region affected the localization of Pal to the outer membrane, we isolated the outer membrane followed by Western blot analysis. The results showed that both truncated variants were properly targeted to the outer membrane, similar to the WT Pal protein (Fig. 1D). This finding extends previous observations that even more extensive deletions in this region (up to 31 residues) still allow proper outer membrane targeting (14).

### Effects on bacterial growth and membrane stability

**Fig 2:**
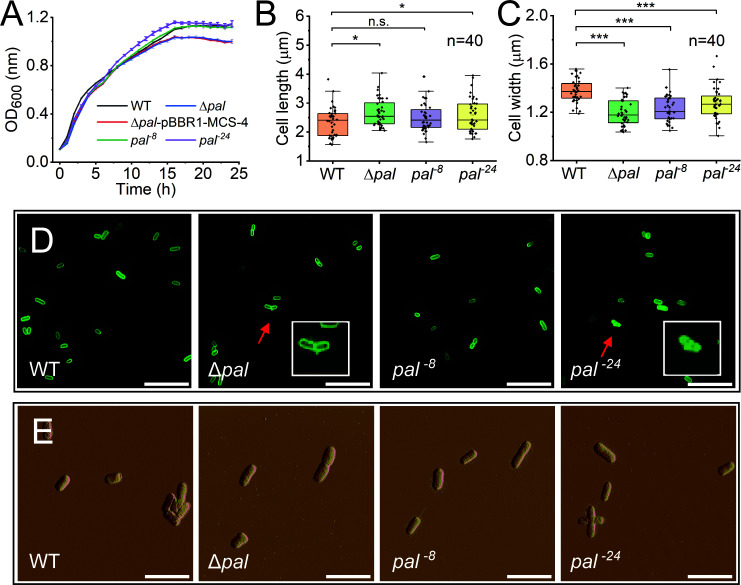
Phenotypic characterization of *E. coli* strains with modified Pal sequences. (**A**) Growth curves of the indicated strains in LB medium at 37°C. Data represent the mean and standard error from three independent experiments. The growth kinetics of the WT, *pal*^−8^, and *pal*^−24^ strains are very similar under these conditions, resulting in closely overlapping curves. (**B and C**) Bacterial cell length (**B**) and width (**C**) measurements derived from fluorescence microscopy images. Data are presented as mean ± SD (*n* = 40 cells per strain). Statistical significance was determined by Student’s *t*-test compared to the WT group. *P < 0.05, ***P < 0.001. n.s., not significant. (**D**) Fluorescence microscopy images of cells stained with the membrane dye FM1-43FX. Scale bar, 10 μm. Insets show magnified views of individual Δ*pal* and *pal*^−24^ cells (arrowheads), highlighting membrane blebbing and vesiculation structures. (**E**) Atomic force microscopy images of bacterial morphology. Scale bar, 5 μm.

We next examined the impact of the disordered region truncation on bacterial growth. In standard LB medium at 37°C, the growth kinetics of all strains were comparable during the exponential phase, with Δ*pal* exhibiting a marginally lower stationary-phase OD_600_ than WT, while *pal*^−8^ and *pal*^−24^ reached final densities indistinguishable from WT (Fig. 2A). Fluorescence microscopy and AFM revealed only minor variations in cell dimensions between mutants and WT (Fig. 2B through E). These results demonstrate that the disordered region is dispensable for normal growth under optimal conditions.

Notably, fluorescence microscopy observations detected occasional vesiculation structures on the cell surface of the Δ*pal* and *pal*^−24^ mutants, whereas no such structures were observed in the WT and *pal*^−8^ strains (Fig. 2D). The vesicular nature of these structures was confirmed by cryo-electron microscopy (Fig. S1). This suggested that complete loss of Pal or extensive truncation of its disordered region (e.g., 24-aa deletion) compromises outer membrane integrity, triggering vesiculation. This observation is consistent with previous reports that linked Pal dysfunction to increased outer membrane vesicle production (16, 17).

**Fig 3:**
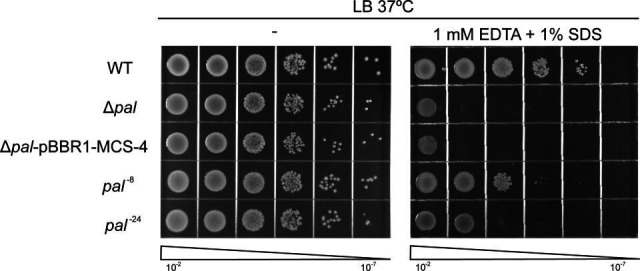
Progressive disruption of outer membrane integrity in *pal* mutants. Growth sensitivity of wild-type (WT), *pal*^−8^, *pal*^−24^, and Δ*pal* strains to membrane-disrupting conditions (1 mM EDTA + 1% SDS). Serial dilutions of cultures were spotted on LB agar plates with or without stressors, then incubated at 37°C. The Δ*pal* mutant showed extreme sensitivity, while *pal*^−8^ and *pal*^−24^ exhibited intermediate but distinct susceptibility (*pal*^−24^ > *pal*^−8^), indicating a dose-dependent role of the disordered region in maintaining membrane stability.

When challenged with membrane-disrupting agents (1 mM EDTA + 1% SDS), the mutants exhibited a graded sensitivity phenotype: compared to the WT, the *pal*^−8^ strain showed increased sensitivity, which was further enhanced in the *pal*^−24^ mutant and was most severe in the Δ*pal* knockout (Fig. 3). This progressive impairment of membrane stability implies that the disordered region contributes incrementally to outer membrane integrity, with its complete absence (Δ*pal*) causing the most severe defect.

### Impact on cell division and Lpp organization

**Fig 4:**
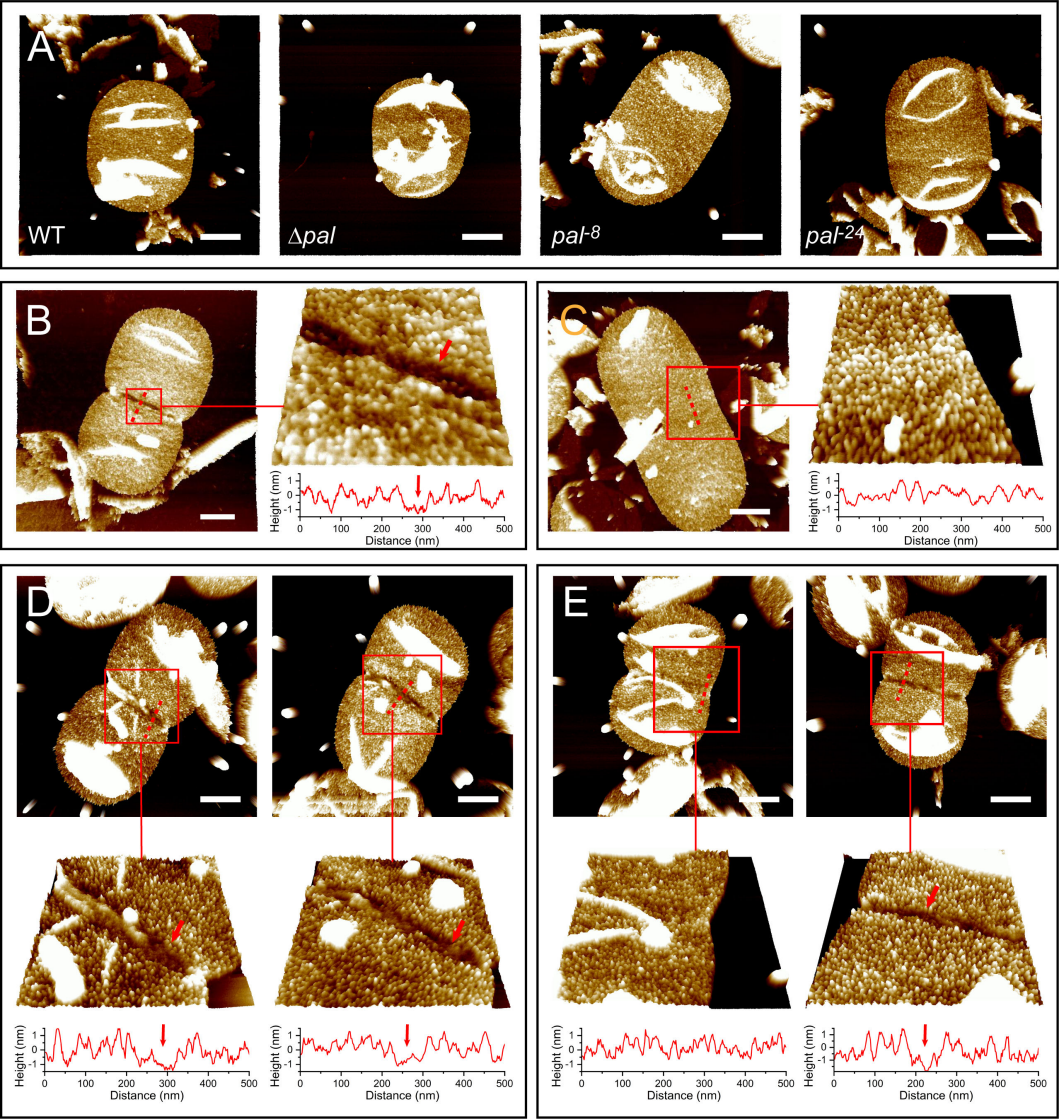
The N-terminal disordered region of Pal is critical for excluding Lpp from the division site. (**A**) AFM images of peptidoglycan sacculi from non-dividing cells of the indicated strains, showing uniform distribution of Lpp particles (high-density bumps) across the surface. (**B–E**) AFM images of sacculi from dividing cells. (**B**) Wild-type (WT) strain, exhibiting a well-defined Lpp-exclusion zone (arrowheads) at the constriction site. (**C**) Δ*pal* mutant, which completely lacks a defined Lpp-exclusion zone. (**D**) *pal*^−8^ mutant maintains a wild-type-like Lpp-exclusion zone (arrows). (**E**) *pal*^−24^ mutant exhibits impaired Lpp exclusion, showing either absent (left) or narrowed (right, arrows) grooves. Scale bars: 500 nm. Red boxes indicate the regions magnified in the adjacent panels. Line profiles beneath the magnified images represent surface height cross-sections along the dashed lines, illustrating the topography of the exclusion groove.

Our most revealing findings emerged from high-resolution imaging of cell envelope dynamics. Using atomic force microscopy to examine the isolated sacculi, we first confirmed that in non-dividing cells, all strains showed equivalent distributions of Lpp particles across the sacculus surface (Fig. 4A). Quantification of the Lpp surface density confirmed that the truncations did not globally disrupt Lpp organization in non-dividing cells (Fig. S2).

However, during cell division, we discovered important differences. The wild-type strain consistently formed a well-defined Lpp-exclusion zone at the constriction site (Fig. 4B). In contrast, the Δ*pal* mutant completely lacked this zone (Fig. 4C). The *pal*^−8^ mutant phenocopied the wild type, maintaining a clear exclusion zone (Fig. 4D), whereas the *pal*^−24^ mutant exhibited an intermediate and variable phenotype (Fig. 4E). While a majority of *pal*^−24^ cells formed an exclusion zone, a substantial fraction displayed incomplete or absent zone formation (Fig. 4E). In those sacculi where a zone was detectable, it was significantly narrower than the exclusion zones in both wild-type and *pal*^−8^ cells, as confirmed by quantitative analysis (Fig. S3). These observations demonstrate that the length of the disordered region fine-tunes the efficiency of spatial exclusion between Pal and Lpp at the division site.

## DISCUSSION

The N-terminal disordered regions found in *E. coli* outer membrane lipoproteins are known to be critical for protein sorting (14, 18), yet their functional roles after successful localization remain largely unexplored. By systematically truncating this region in Pal, we uncover a length-dependent mechanism that operates during cell division. Specifically, the disordered linker acts as a molecular spacer that fine-tunes the spatial competition between Pal and Lpp at the division site. This competition ensures the formation of an Lpp-free zone, which contributes to efficient outer membrane remodeling (9). Our work thus reveals a length-governed function for a disordered linker in coordinating a key step of bacterial cell division.

Our findings that shorter linkers lead to graded defects in Lpp exclusion and membrane integrity prompt the question of how linker length influences Pal function. Given that Pal is anchored in the outer membrane and must bridge the periplasm to interact with the peptidoglycan layer (14, 19), the disordered linker likely serves as a flexible tether whose length is critical for productive engagement. Specifically, we speculate that a linker of sufficient length is required for two distinct interactions. First, it may allow the C-terminal peptidoglycan-binding domain to physically reach and engage the peptidoglycan layer at the division site (10). Excessively shortening the linker could sterically hinder this interaction, thereby diminishing Pal’s competitive advantage over Lpp at the division site. Second, and equally important, Pal is a core component of the Tol-Pal system, and its dynamic recruitment to the division site involves interaction with other system components (6, 7). An adequately long, flexible linker may be necessary to permit the conformational changes or precise positioning required for these intra-system interactions to occur efficiently. A truncated linker might constrain these dynamics, impairing Pal’s integration into the functional Tol-Pal complex at the septum. The critical importance of linker length for bridging spatial gaps in the envelope finds strong precedent in other systems. For instance, increasing the periplasmic distance by lengthening Lpp impaired RcsF’s ability to interact with its inner membrane partner, IgaA, unless RcsF itself was extended (20, 21). Similarly, the N-terminal flexible linker of LpoB is vital for its *in vivo* function, as truncation abolishes its ability to reach and activate PBP1B despite retaining normal binding affinity *in vitro* (22). These examples underscore a common principle: disordered linkers can act as critical spacers, enabling proteins to span specific distances and function effectively within the constrained geometry of the cell envelope. By analogy, the length of Pal’s disordered region may be similarly tuned to ensure it can simultaneously fulfill its dual structural and partnership roles during cell division.

In considering these possibilities, two underlying questions warrant examination. First, the functional defects we observe presuppose that the truncated Pal variants are correctly delivered to the outer membrane. While we cannot entirely exclude minor alterations in trafficking, even the more severely truncated Pal_Δ26–56_ variant was shown to localize predominantly to the outer membrane (14). It is therefore reasonable to infer that our less extensive truncations also achieve efficient outer membrane targeting, suggesting the phenotypes arise from functional deficiencies of properly localized protein. Second, our model hinges on the idea that linker length affects the efficiency of Pal’s engagement with its targets rather than abolishing its intrinsic binding capacity. This view is supported by the preserved structure of the globular C-terminal peptidoglycan-binding domain in our AlphaFold predictions and by the spatial separation between our truncations and this domain. Thus, the intrinsic binding affinity of Pal’s C-terminal domain is likely intact. A more plausible explanation is that shorter linkers perturb the spatial reach or interaction dynamics of the protein within the cell.

Several methodological and experimental considerations help to define the context of our findings. First, our AFM analysis specifically examined peptidoglycan sacculi at the initial stage of constriction. This focus was a deliberate choice because sacculi from later division stages frequently develop folds and wrinkles during sample preparation, which obscure the Lpp-exclusion zone and preclude reliable measurement (9). By analyzing only early-stage sacculi, we ensured that comparisons across strains were quantitative and unbiased by such imaging artifacts. Second, regarding protein expression, our Pal truncation variants were expressed from a plasmid, whereas the wild-type protein is chromosomally encoded. This difference in genetic context introduces the possibility that protein abundance could vary between strains, which in principle might influence the observed phenotypes. However, the clear, graded phenotypic differences between the *pal*^−8^ and *pal*^−24^ mutants, both expressed from the same plasmid backbone, demonstrate that linker length is a primary determinant of function, irrespective of potential absolute expression-level differences from the wild type. Together, these considerations delineate the specific conditions under which our conclusions are drawn and reinforce the robustness of the link between linker length and functional output.

Several questions remain that could be addressed in future work. A key mechanistic issue is whether the disordered nature of the linker itself, apart from its length, is functionally important. This could be tested by replacing the native disordered sequence with a structured α-helix of similar length, an approach successfully used to dissect linker function in lipoproteins (14). Furthermore, our attempts to construct a Pal_Δ26–61_ mutant (36-residue deletion) were unsuccessful, with no detectable protein expression. This technical challenge mirrors El Rayes et al.’s reported inability to detect their Pal_Δ25–68_ construct (44-residue deletion) (14). These consistent results suggest that the disordered linker cannot be arbitrarily shortened below a critical length threshold without compromising protein stability or function. Future studies could explore whether a minimal linker length is required for Pal to function effectively and whether altering this length affects its ability to coordinate outer membrane constriction during cell division.

In clades such as the Enterobacterales and other related gammaproteobacteria that encode both Pal and Lpp (5), the presence of a disordered region in Pal and its role in mediating spatial competition with Lpp have broader implications for bacterial physiology and evolution. Disordered regions are known to confer functional versatility to proteins, allowing them to adapt to changing environmental conditions. In the case of Pal, the disordered region may enable the protein to dynamically interact with the peptidoglycan layer and other components of the cell envelope, ensuring robust cell division under diverse conditions. Furthermore, the compensatory relationship between Pal and Lpp highlights the evolutionary flexibility of these bacteria in maintaining essential cellular processes.

## MATERIALS AND METHODS

### Bacterial strains, plasmids, and growth conditions

All strains and plasmids used in this study are listed in Table S1. *E. coli* strains were grown in LB medium (10 g/L tryptone, 5 g/L yeast extract, and 10 g/L NaCl) at 37°C with shaking (180 rpm). When required, ampicillin (100 μg/mL) was added for plasmid maintenance.

### Strain construction

The *pal*^−8^ (ΔK26-S33) and *pal*^−24^ (ΔK26-N49) mutants were constructed by inverse PCR mutagenesis of the *pal* gene in plasmid pBBR1MCS-4 using the QuickChange Lightning Kit (Agilent Technologies). Mutations were verified by sequencing and subsequently introduced into the Δ*pal* strain by electroporation.

### RNA extraction and RT-qPCR

Total RNA was isolated from stationary-phase cultures using the RNeasy Mini Kit (Qiagen) and treated with DNase I. cDNA was synthesized using the High-Capacity cDNA Reverse Transcription Kit (Applied Biosystems). RT-qPCR was performed with TB Green Premix Ex Taq II (Takara) on a QuantStudio 3 System (Applied Biosystems). The *recA* gene was used as an internal control.

### Growth curve analysis

Overnight cultures were diluted 1:100 in fresh LB medium and incubated at 37°C. Growth kinetics was monitored by measuring optical density at 600 nm using a BioScreen C automated growth curve analyzer. Three biological replicates were performed for each strain.

### Membrane fractionation and Western blotting

Outer membrane fractions were isolated by sucrose density gradient centrifugation as previously described (23). Briefly, cells were lysed by passage through a high-pressure cell disrupter, and total membranes were separated on a 20%/53%/73% sucrose gradient by ultracentrifugation (65,000 × *g*, 18 h). Outer membrane fractions were confirmed by SDS-PAGE and probed with anti-Pal antiserum (1:4,000 dilution).

### Fluorescence microscopy

Cells were labeled with the membrane dye FM1-43FX (ex/em = 480/580 nm, Thermo Fisher Scientific) at a final concentration of 10 μg/mL for 10 min. Images were acquired using a Nikon Eclipse Ti microscope equipped with a ×100 oil immersion objective and a standard GFP filter set.

### Peptidoglycan sacculi preparation

Peptidoglycan sacculi were prepared as described (9). Cells were collected by centrifugation (3,000 × *g*, 5 min) and washed three times with distilled water. The pellet was resuspended in distilled water and lysed by passage through a high-pressure cell disrupter (Constant Systems Ltd). The suspension was centrifuged (3,000 × *g*, 5 min) to remove unbroken cells, and the supernatant was subsequently centrifuged at 20,800 × *g* for 60 min to collect the sacculi. The pellet was resuspended in 5% (wt/vol) SDS and boiled for 60 min. The SDS-insoluble sacculi were washed with Milli-Q water by ultracentrifugation (156,000 × *g*, 20 min) at least three times. The final pellet was suspended in Milli-Q water for AFM imaging.

### Atomic force microscopy

A 1.5 μL drop of the sacculi suspension was deposited onto freshly cleaved mica and air-dried. AFM imaging was performed using a Multimode VIII AFM with a Nanoscope V controller (Bruker) in ScanAsyst mode in air. Silicon cantilevers (XSC11/AI BS, MikroMasch) were used. Section analyses were performed using NanoScope Analysis v.1.40 software. For the analysis of Lpp-exclusion zones, only sacculi representing the initial stage of constriction were analyzed. This criterion was applied because sacculi from cells at later division stages are highly prone to folding artifacts at the deep constriction site during air-drying, which obscure the exclusion zone and prevent reliable measurement (9).

### Membrane integrity assays

Membrane integrity was assessed using a spot assay based on exposure to EDTA and SDS, as described elsewhere (24, 25) with the following specifics. Five microliters of serial 10-fold dilutions of cultures was spotted onto LB agar plates containing 1 mM EDTA and 1% SDS. Plates were incubated at 37°C for 16 h before imaging.

### Statistical analysis

All experiments were performed with at least three biological replicates. Data are presented as mean ± standard deviation. Statistical significance was determined using Student’s *t*-test.

## SUPPLEMENTAL MATERIAL

Supplemental figures and table jb.00408-25-s0001.pdf Figures S1 to S3 and Table S1.

ASM does not own the copyrights to Supplemental Material that may be linked to, or accessed through, an article. The authors have granted ASM a non-exclusive, world-wide license to publish the Supplemental Material files. Please contact the corresponding author directly for reuse.
