# Supplementary material for: Length matters: the disordered N-terminus of Pal coordinates Lpp exclusion for outer membrane constriction in E. coli
Source: J Bacteriol. 2025 Dec 23;208(1):e00408-25. doi: 10.1128/jb.00408-25 (PMC12826056; doi:10.1128/jb.00408-25)
Supplement: Supplemental figures and table — Figures S1 to S3 and Table S1. [file jb.00408-25-s0001.pdf]

Supplementary Materials for

Length Matters: The Disordered N-terminus of Pal Coordinates Lpp Exclusion for Outer Membrane Constriction in *E. coli*

Zhuo-Wei Chen, Ting-Ting Chen, Hong-Su Zhang, Si-Yu Chen, Yu-Qing Zhang, Xiu-Lan Chen, Yu-Zhong Zhang, Hai-Nan Su*

*Corresponding author: suhn@sdu.edu.cn (H.N.S.)

This PDF file includes:

Figs. S1 to S3

Supplementary Table 1

Supplementary Figures

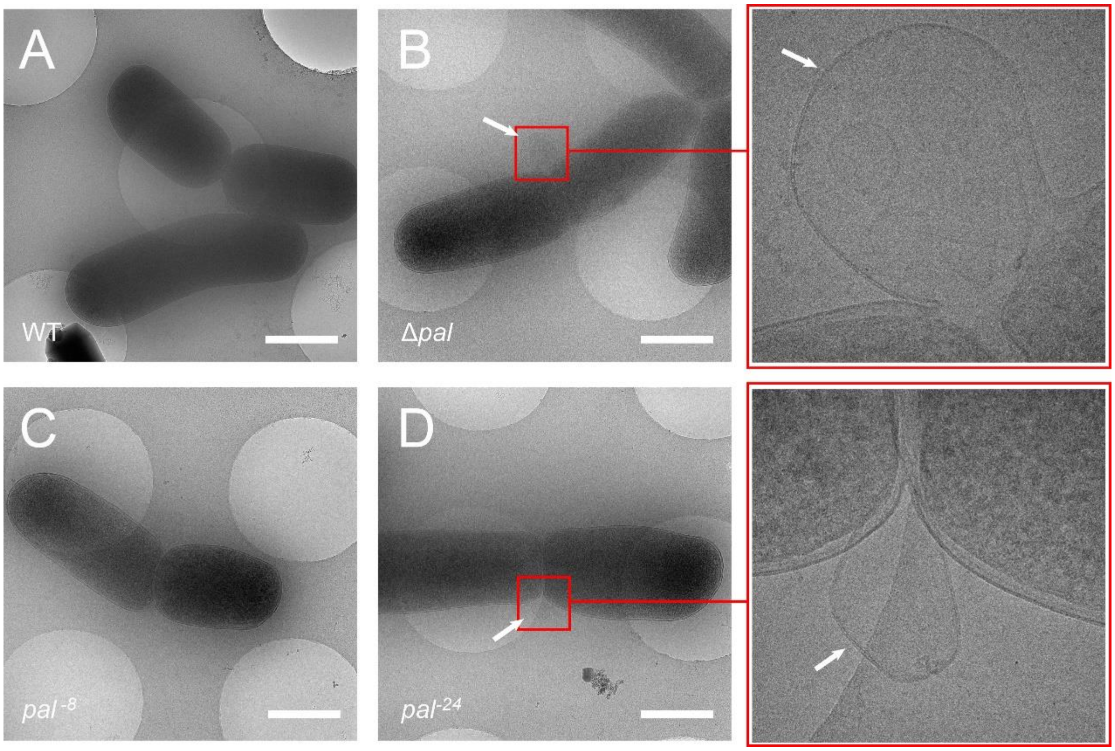

Fig. S1. Cryo-electron microscopy (cryo-EM) analysis of outer membrane vesiculation in *E. coli* strains. (A-D) Representative cryo-EM images of whole cells for the indicated strains. (A) Wild-type (WT) cell. (B) Δ*pal* mutant cell. (C) *pal*$^{-8}$ mutant cell. (D) *pal*$^{-24}$ mutant cell. Scale bars: 1 μm. The red boxes in (B) and (D) indicate the areas magnified to show budding outer membrane vesicles (OMVs, white arrow). Panels at the right of (B) and (D) show high-magnification views of the boxed region.

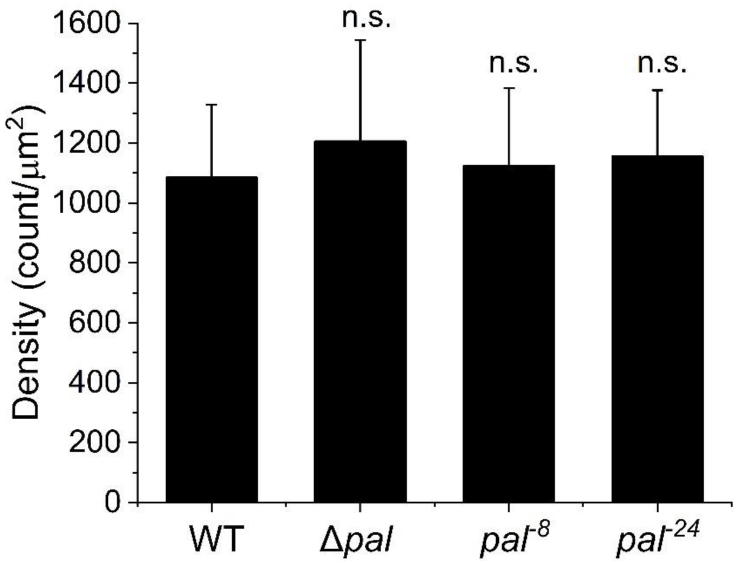

Fig. S2. Quantification of Lpp surface density on peptidoglycan sacculi. Surface density of Lpp particles (number of particles per μm²) was measured on sacculi from the indicated strains in non-dividing cells. Data are presented as mean ± standard deviation (n ⩾ 10 sacculi per strain). Statistical analysis was performed using student's t-test, comparing each mutant to the WT strain. n.s., not significant.

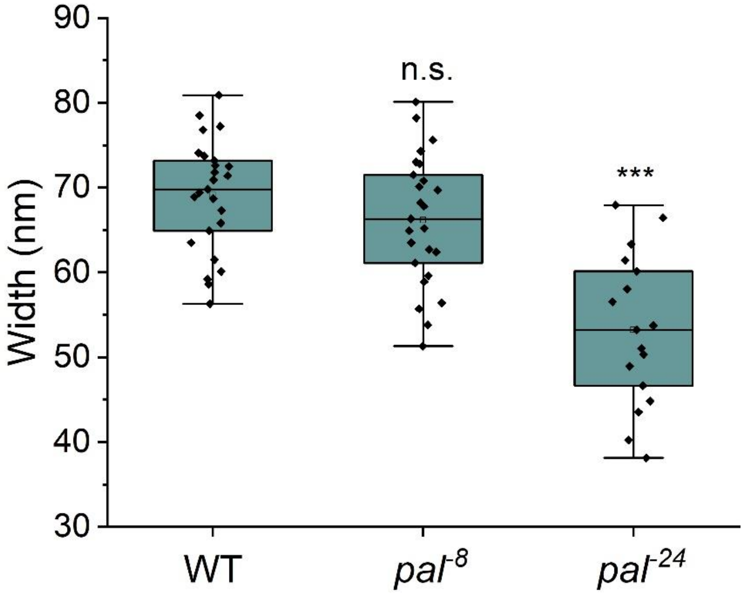

Fig. S3. Quantification of Lpp-exclusion zone width at division sites. Box-and-whisker plots show the distribution of Lpp-exclusion zone widths measured from AFM images of dividing sacculi for the indicated strains. Each data point corresponds to one measured exclusion zone from an individual sacculus (WT, n = 25; *pal*$^{-8}$, n = 25; *pal*$^{-24}$, n = 17). Statistical significance was determined by student's t-test, comparing each mutant to the WT strain. *** $p < 0.001$, n.s., not significant.

Supplementary Table 1. Bacterial strains used in this study

Species	Description	Source
E. coli MG1655	Wild-type	Lab stock
Δ*pal*	MG1655 with *pal* gene deletion	Lab stock
pal^{-8}	MG1655 with 8-aa deletion (K26-S33) in Pal	This study
pal^{-24}	MG1655 with 24-aa deletion (K26-N49) in Pal	This study
